# The association between allergic rhinitis and otitis media: A national representative sample of in South Korean children

**DOI:** 10.1038/s41598-018-38369-7

**Published:** 2019-02-07

**Authors:** Haewon Byeon

**Affiliations:** 1 Department of Speech Language Pathology, College of Health Science, Honam University, Gwangju, Republic of Korea; 2 Present Address: Department of Speech Language Pathology, School of Public Health, Honam University, 417, Eodeung-daero, Gwangsan-gu, Gwangju, Republic of Korea

## Abstract

Although many studies have reported that allergic rhinitis is an independent risk factor highly related to otitis media in children, there is still lack of epidemiological studies on demographics. The objective of this study was to identify if allergic rhinitis was an independent risk factor for otitis media in children aged between 7 and 12 years by using the nationwide survey data representing the local population of South Korea. This is a secondary study based on the ENT examination data (eg. acute otitis media, otitis media with effusion, chronic otitis media). The subjects of this study were 472 children (248 male and 224 female) who completed the Korea National Health and Nutrition Examination Survey 2015. The presence of otitis media was examined by otolaryngologists using tympanometric measurements, audiometric measurements, and otoscopic examination. Allergic rhinitis was diagnosed by Korean-version of International Study of Asthma and Allergies in Childhood, a total serum immunoglobulin E test, an allergen-specific immunoglobulin E test, a blood eosinophil test, an eosinophil cationic protein test, a nasal cytology for eosinophils test, a skin reaction test, and an antigen simultaneous test. Confounding factors included age, gender, the levels of income for households, and household composition. The relationship between allergic rhinitis and otitis media was analyzed by a complex sample logistic regression analysis and the odds ratio and 95% confidence interval were presented. The results of a complex sample design logistic regression revealed that allergic rhinitis in children was significantly associated with otitis media (p < 0.05). Even after adjusting all confounding factors, children with allergic rhinitis had twice significantly higher risk of otitis (OR = 2.04; 95% CI: 1.30–3.18) than children without allergic rhinitis. This epidemiologic study confirmed the independent relationship between pediatric allergic rhinitis and otitis media. In the future, longitudinal study will be needed to verify causality of allergic rhinitis and otitis media.

## Introduction

Otitis media is an inflammation of the ear that is caused by bacterial infiltration into the tympanic cavity. It is a very common ear illness to young children. Otitis media is low in prevalence in newborns but is most prevalent around the age of 2^1^ and it is Estimated that death rate related to otitis media is especially high under the age of 5^2^. Thus, epidemiological studies on the prevalence of otitis media have mainly reported the results of tests on infants and young children under the age of 2^3^ and there are only a few epidemiological studies which reported the prevalence of school-age children^4^. Previous epidemiological studies reported that the prevalence of otitis media in children under 14 years old ranged between 3.7 and 8.7%^5–10^. It was revealed that the prevalence of otitis media in developing countries is higher than in developed countries^8^.

Otitis media is likely to become a chronic disease if it is not proper treatment is in childhood due to frequent recurrences. A previous study reported that five out of 100 young children suffered from recurrent otitis media^11^. In the United States, the recurrence rate of otitis media in preschool children is steadily increasing^12^. Otitis media is a major reason for the doctor’s visit in South Korean children as well. As of 2008, otitis media is the sixth most common cause of the doctor’s visit for patients under 10 years old^13^. Since the childhood health is known to have important effects on the health of adolescents and adulthood, it is required to pay attention to otitis media of children to maintain healthy hearing throughout life.

Although otitis media is not a life-threatening illness, chronic otitis media can adversely affect language development, such as speaking and listening, which can decrease learning performance^12,14^. In severe cases, it can lead to complications such as hearing loss and degrade the quality of life^12,14^. Therefore, identifying the risk factors of otitis media and managing them in the early stage are important from the prophylactic point of view.

Previous studies indicated that age, gender, race, genetic factors, low socioeconomic status, breastfeeding, allergic diseases, and chronic sinusitis were associated with otitis media^6,7,15,16^. Several studies of them reported that allergic rhinitis was a risk factor affecting the occurrence of otitis media^17^. Kim *et al*.^18^ in their case-control study revealed that allergic rhinitis and day nursery were independent risk factors highly associated with young children’s otitis media. Risk factors can be divided into factors that cannot be changed such as genetic factors and family history and factors that can be changed such as environment and lifestyle. The risk and recurrence of otitis media can be reduced by identifying and managing these changeable factors.

Although allergic rhinitis has been reported as an independent risk factor of otitis media, the most of the previous studies targeted only subjects visiting a specific medical facility or were conducted as case-control studies based on a small sample size. Consequently, it is limited to generalize the obtained results to the population of the local community^18–20^. Moreover, studies evaluated the illness of young children under 5 years old identified the history of otitis media using a parental questionnaire^12^. Consequently, these results pose a possibility to suffer from the bias due to the limited remembrance^12^. Furthermore, there are only few studies that were adjusted for considering the environmental factors such as the household economy level and day nursery care in addition to the sociodemographic factors. Although environmental, racial, and sociocultural differences are highly associated with the risk factors of otitis media, few epidemiological studies have investigated the risk factors of otitis media in young South Korean children. More epidemiological studies are needed to develop an evidence-based otitis media guideline because the risk factors for an illness can vary by culture and race.

Therefore, it is necessary to conduct an epidemiological study targeting a population in order to identify the risk factors of young children’s otitis media in South Korea. The objective of this study was to identify if allergic rhinitis was an independent risk factor for otitis media in children aged between 7 and 12 years by using the nationwide survey data representing the local population of South Korea.

## Methods

### Data Source and Participants

The source of this study is Korea National Health and Nutrition Examination Survey (KNHNES) conducted by Korea Centers for Disease Control and Prevention in 2015 with the support of the Ministry of Health and Welfare for the purpose of calculating representative and reliable statistics for the health level and health behavior of South Koreans. KNHANES was approved by the Institutional Review Board of the Korean Center for Disease Control and Prevention (2013-12EXP-03-5C) and carried out compliant with the ethical standards of the Declaration of Helsinki. Source data were provided to the researchers by data management personnel of Korea Center for Disease Control in pseudonymized condition with yet other virtual IDs attached after personal information being deleted. The survey procedures were designed to protect participant privacy by allowing anonymous and voluntary participation. Participants were given identification numbers and guaranteed anonymity. After the survey had been fully explained and all participants had provided written informed consent (both directly and from their parents or legal guardians), participants completed a survey. Authors reported the objective of the study to Korea Centers for Disease Control and Prevention and obtained an official permit to analyze the raw data. KNHANES is based on a rolling sampling design that employs a complex, stratified multistage probability cluster survey of a representative sample of the non-institutionalized population in South Korea (please refer^6^ for further detail of the sampling methods of the KNHANES). Briefly, KNHANES in 2015 was carried out for 9,773 subjects from 3,840 households and the response rate was 78.3% (n = 7,649).

**Figure 1:**
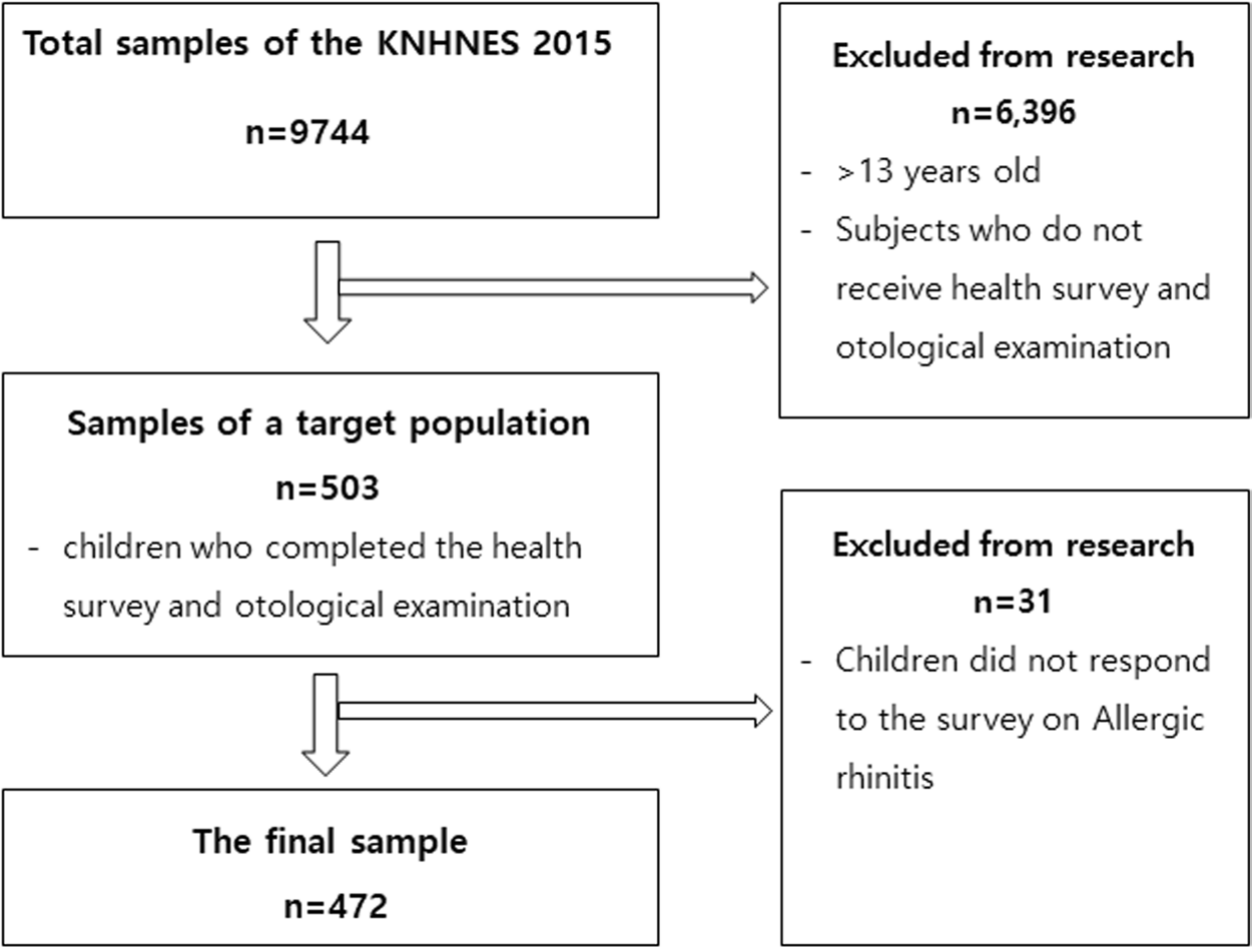
Flow chart.

The subjects of this study were 503 children who completed the health survey and otological examination. Among these subjects, 31 children did not respond to the survey on Allergic rhinitis so they were excluded from the study. Consequently, 472 children (248 male and 224 female) were used for further analyses. The flow chart is shown in Fig. 1.

### Measurement

#### Health interview

Trained medical staffs conducted the otological examination at a mobile examination center and the health interview at participants’ homes. For the health interview, the information regarding the highest level of education and economic activities was obtained from face-to-face interviews and the survey on behaviors associated personal health was carried out by using self-administered questionnaires.

#### Otitis media and allergic rhinitis

The otolaryngological examinations were conducted by following the technical advice provided by The Korean Otologic Society and highly trained 40 otolaryngologists^10^. Otitis media was diagnosed by the following procedure. As a first step, the swelling, blister formation, redness, perforated eardrum associated with otorrhea, the presence of middle ear effusion, and the color changes in eardrum were checked through direct observation using otoscopy. Afterward, type B or type C middle ear effusion was checked using the tympanometry test. Patients with Down syndrome, palatal facial deformities such as cleft palate, immune disorders, and cochlear implants were excluded from the test. Allergic rhinitis was diagnosed by conducting a total serum immunoglobulin E test, an allergen-specific immunoglobulin E test, a blood eosinophil test, an eosinophil cationic protein test, a nasal cytology for eosinophils test, a skin reaction test, and an antigen simultaneous test. This is a secondary study based on the ENT examination data. Individual diagnostic data (primary data) researched by ENT doctors were provided after being converted into binary data (e.g. otitis media – yes, no) in KNHNES.

#### Confounding factors

Age, gender, the levels of income for households, and household composition were examined. The levels of income for households were classified into four quartiles. Household composition was classified into two generations households composed of married couples and children, two generations households composed of single parents and children, and three generations households.

### Statistical Analysis

The weighted values of KNHANES were calculated so that the subjects of the survey can represent overall Korean population. Detailed explanations on the weighted values are shown in reference^10^.

The general characteristics of the subjects and the prevalence of otitis media were presented as weighted percentages. Differences in the groups by the prevalence of allergic diseases were confirmed by Rao-Scott Chi-square test. The relationship between allergic rhinitis and otitis media was analyzed by a complex sample logistic regression analysis and the odds ratio and 95% confidence interval were presented. Gender and age were adjusted in the first level of the study model and all confounding factors including household economic level and household composition were adjusted in the second level additionally. IBM SPSS version 24.0 (IBM Inc., Chicago, Illinois) was used for analyses and significance level was 0.05 unless stated otherwise.

## Results

### The general characteristics of the study subjects

The general characteristics of the study subjects are presented in Table 1. The mean age was 9.6 years (standard error = 0.4) and there were more male subjects (50.5%) than female subjects (49.5%). The fourth quartile household economy level was the most common (33.4%) and the two generations households, which were composed of married couples and children, were dominant (75.3%). The prevalence of allergic rhinitis was 26.5% and the prevalence of otitis media was 26.6%.

**Table 1:** The general characteristics of the study population, Weighted %.

Characteristics	Weighted % ± SE
Age, Weighted Mean ± SE	9.6 ± 0.4
Gender	
Male	50.5 ± 2.4
Female	49.5 ± 2.4
Levels of income for households	
1^st^ quartile	9.8 ± 1.7
2^nd^ quartile	25.8 ± 3.3
3^rd^ quartile	31.7 ± 2.9
4^th^ quartile	33.4 ± 3.6
Household composition	
Two generations households composed of married couples and children	75.3 ± 2.6
Two generations households composed of single parents and children	13.4 ± 1.8
Three generations households	11.3 ± 1.7
Otitis media	
No	73.4 ± 2.3
Yes	26.6 ± 2.3
Allergic rhinitis	
No	73.5 ± 2.4
Yes	26.5 ± 2.4

### The general characteristics of the subjects according to the prevalence of otitis media

The general characteristics of the subjects according to the prevalence of otitis media in young children are shown in Table 2. The results of Rao-Scott Chi-square test showed that the prevalence of otitis media was significantly (p < 0.05) higher in children with allergic rhinitis (37.2%) than in those without any allergic rhinitis (22.7%), which was an only significant variable.

**Table 2:** The characteristics of the subjects according to the prevalence of otitis media: Rao-Scott Chi-square test, Weighted %.

Characteristics	Otitis media		*p*
	No (n = 346)	Yes (n = 126)	
Gender			0.802
Male	73.8	26.2	
Female	72.8	27.2	
Age			0.311
7	73.2	26.8	
8	67.1	32.9	
9	81.4	18.6	
10	79.4	20.6	
11	71.3	28.7	
12	69.8	30.2	
Levels of income for households			0.077
1^st^ quartile	88.0	12.0	
2^nd^ quartile	71.8	28.2	
3^rd^ quartile	69.3	30.7	
4^th^ quartile	73.3	26.7	
Household composition			0.102
Two generations households composed of married couples and children	70.9	29.1	
Two generations households composed of single parents and children	77.6	22.4	
Three generations households	82.0	18.0	
Allergic rhinitis			0.002
No	77.3	22.7	
Yes	62.8	37.2	

### The relationship between allergic rhinitis and otitis media

The relationship between allergic rhinitis and otitis media in young children is shown in Table 3. The results of a complex sample design logistic regression revealed that allergic rhinitis in children was significantly (p < 0.05) associated with otitis media. Even after adjusting all confounding factors (Model 2), children with allergic rhinitis had twice significantly (p < 0.05) higher risk of otitis (OR = 2.04; 95% CI: 1.30–3.18) than children without allergic rhinitis.

**Table 3:** The relationship between allergic rhinitis and otitis media in young children: complex sample design logistic regression, odds ratio (95% CI).

Allergic rhinitis	Unadjusted	Model 1	Model 2
No	1	1	1
Yes	2.01 (1.30, 3.12)*	2.02 (1.31, 3.13)*	2.01 (1.29, 3.15)*

*p < 0.05

Model 1: Adjusted for age, gender.

Model 2: Additionally levels of income for households.

Model 3: Additionally adjusted for household composition.

## Discussion

This epidemiological study examined the relationship between allergic rhinitis and otitis media in children from 7 to 12 years old in South Korea. The results of this study showed that the prevalence of otitis media in children between 7 and 12 years old was 26.6%. Previous studies on the prevalence of otitis media in children were conducted in a small area. Although there was a small variation in the reported prevalence of otitis media in preschool children, previous studies reported high prevalence of otitis media in these children, which agreed with the results of this study: 16.4% for Daegu^21^, 15.8% for Bucheon^22^, and 14.5% for Gunsan^23^. Even though it is difficult to compare directly, the prevalence of this study was higher than the prevalence of otitis media with effusion (10.4%) in children between 6 and 11 years old (2,355 people) in Eastern Anatolia^16^, the prevalence of otitis media with effusion (8.7%) in 1,800 children registered primary schools in Istanbul^24^ and the prevalence of otitis media with effusion (10.5%) in 5-year-old preschool children in the UK^25^.

Particularly, Pyo *et al*.^26^ examined the prevalence rate of otitis media in South Korean kindergarten and elementary school students (n = 3,364) and they reported that the prevalence was higher during an in-between season (March; 18.5%) than during a season (6.5%). The results of Pyo *et al*.^26^ suggested that the prevalence of otitis media had a seasonal change and it could be affected by the prevalence of seasonal allergic rhinitis^25^. It will be necessary to conduct a nation-wide time series analysis in order to understand the relationship between the seasonal prevalence of otitis media and allergic rhinitis.

The results of this study showed that, even after adjusting confounding factors, children with allergic rhinitis had twice and significantly higher risk suffering from otitis media than those without allergic rhinitis. Many previous case-control studies consistently reported that allergic rhinitis was significantly associated with otitis media, concurring with the results of this study. For example, a case-control study conducted for children between 4 to 12 years old in Hong Kong indicated that children with allergic rhinitis had a 4.6-fold (OR = 4.64) higher risk of otitis media than children without rhinitis^19^. Similarly, a case-control study in children under 60 months revealed that allergic rhinitis had a 2.3-fold higher risk of otitis media (OR = 2.32) even after adjusting for confounding factors^18^. The authors could conclude that there was insufficient evidence that allergic rhinitis directly affected the etiology of otitis media but otitis media in young children could be deteriorated continuously due to respiratory allergies. Similar results were reported in a pediatric epidemiologic study in the United States. The study examined children aged less than 6 years (5,189 people in 1918 and 6,209 people in 1988) participating in the National Health Interview Surveys (NHIS) from 1981 to 1988 and analyzed the risk factors for otitis media to revealed that the increase in allergic rhinitis was significantly related with an increase in otitis media^12^.

It is known that otitis media may be caused by various factors such as dysfunction of the Eustachian tube, inflammation reaction, and atopy. A recent meta-analysis indicated that allergic rhinitis was identified as a significant risk factor for otitis media with effusion^27^. Nevertheless, the scientific mechanism to explain the causal relationship between allergic rhinitis and otitis media is still insufficient^12^. This epidemiological study presents three possible mechanisms to explain the relationship between allergic rhinitis and otitis media in young children.

The first possible mechanism to explain the relationship is “shock organ” hypothesis. The hypothesis speculates that allergic reactions cause mast cells in the mucosa and inflammatory cells in the nose and nasopharynx. They release cytokines and other inflammatory mediators resulting in the occlusion of Eustachian tubes to develop otitis media^17,28^.

The second hypothesis is the dysfunction of Eustachian tubes as a cause of otitis media. It means that the occlusion of Eustachian tubes disturbs the ventilation in the middle ear cavities to increase the absorption of nitrogen in the middle ear, produce continuous negative pressure, and result in the occurrence of otitis media^17^. The temporary dysfunction of Eustachian tubes is known to cause the aspiration into the middle ear cavity of nasopharyngeal secretions containing bacteria and viruses. This dysfunction of Eustachian tubes is known to be frequent in young children with allergic rhinitis^29,30^.

The third hypothesis is the degradation of the immune function. Children have relatively weaker immune functions than adults. Particularly, it is believed that young children with allergic rhinitis are at higher risk of allergic otitis because young children with allergic rhinitis experience the dysfunction of Eustachian tubes more frequently due to the inflammation of lymphoid tissues such as adenoids. Longitudinal studies are needed to understand causality of allergic rhinitis and otitis media in the future.

Since young children with allergic rhinitis are at high risk for otitis media, it is important to prevent the aggravation of allergic rhinitis by managing risks systematically in order to prevent complications (e.g., otitis media). Many countries have prepared an evidence-based guideline for otitis media. The 2004 evidence-based clinical practice guideline published by Agency for Healthcare Research and Quality (AHRQ) recommended doctors to reduce the risk factors of otitis media for children between 2 months and 12 years old in order to prevent the occurrence of otitis media^31^. Moreover, Ovetchkine and Cohen^32^ emphasized that the treating the nasal cavity by using antihistamines was helpful to treat otitis media when young children with otitis media accompanies with nasal diseases such as allergic rhinitis or sinusitis. In South Korea, The Korean Otologic Society developed the clinical practice guideline for infant and children with otitis media in 2009 for treating young children based on evidence. The society also published a revised version of The Practice Guideline for Pediatric Otitis Media in 2014 with supplementing the results of evidence-based studies^33^. The Practice Guideline for Pediatric Otitis Media emphasized the infection factors, genetic factors, socioeconomic factors, and environmental factors as risk factors for pediatric otitis media. However, it is still lacking information regarding related diseases such as allergic rhinitis that can aggravate otitis media. It is necessary to revise the guidelines for the prevention of otitis media, which contains additional evidence for the deterioration factors of otitis media in young children.

The importance of this study was that this study confirmed the relationship between allergic rhinitis and otitis media in young children by using representative epidemiological data of South Korea. The presented study has four main limitations. First, this study only identified the relationship between the prevalence of allergic rhinitis and otitis media. In the future, it is necessary to evaluate the dose-response relationship between the duration of allergic rhinitis and the degree of otitis media. Secondly, subtypes of otitis media were not investigated. It will be necessary to classify otitis media into subtypes (e.g., otitis media with effusion and suppurative otitis media) and identify the relationship between the subtype and allergic rhinitis. Thirdly, it is possible that there are more confounding factors of otitis media in addition to the confounding factors considered in the study. Future studies are required to control more potential confounding factors such as genetic factors, family history factors, and environmental sanitation factors (ex. secondhand smoke exposure, daycare attendance, urban vs rural setting). Fourthly, it is difficult to find a causal relationship in this study because it was a cross-sectional study. A longitudinal study will be needed to verify causality.

## Conclusion

This population-based epidemiologic study confirmed the independent relationship between pediatric allergic rhinitis and otitis media. The results of this epidemiologic study ask young children, who are suffering from allergic rhinitis to take regular otologic tests such as eardrum test and hearing test and manage rhinitis for preventing otitis media because they are at high risk for complications of otitis media in the future.
